# Long term whole body vibration training has no effects on plantar foot sensitivity and balance control

**DOI:** 10.1186/1757-1146-5-S1-O22

**Published:** 2012-04-10

**Authors:** Günther Schlee, Thomas L Milani

**Affiliations:** 1 Department of Human Locomotion, Chemnitz University of Technology, Chemnitz, 09126, Germany

## Background

Below-threshold vibration together with low-level mechanical noise (stochastic resonance) is known to have positive effects on plantar foot sensitivity and balance control [1]. However, the effects of above-threshold stimulation on both variables are still not proved. The goal of this study was to investigate the effects of whole body vibration (WBV) training, characterized by above threshold stimulation, on plantar foot sensitivity and balance control of young healthy subjects.

## Materials and methods

38 subjects of both genders were divided in training (WBV, n=27) and control (CG, n=11) groups. Plantar foot vibration sensitivity and balance were measured before and after a 6-week WBV training, in which subjects were exposed weekly to three bouts of vibration stimuli (27 Hz vibration frequency; 2 mm horizontal amplitude), with duration from 5.30 up to 8.30 min. Vibration sensitivity was measured at the heel, first and fifth metatarsal heads and hallux of both feet. Balance was measured with subjects standing on one leg (right and left legs) during 20 s with eyes open. Vibration thresholds [µm] and CoP excursion [mm] before and after training were compared with a Wilcoxon Test (α=.05).

## Results

**Figure 1:**
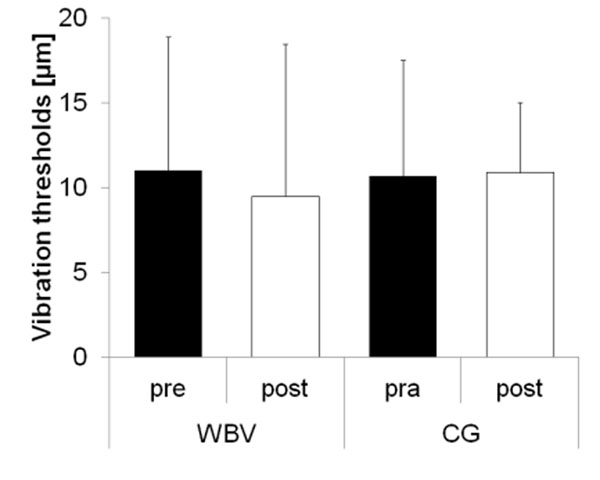
Thresholds [µm] at the Hallux: right foot

**Figure 2:**
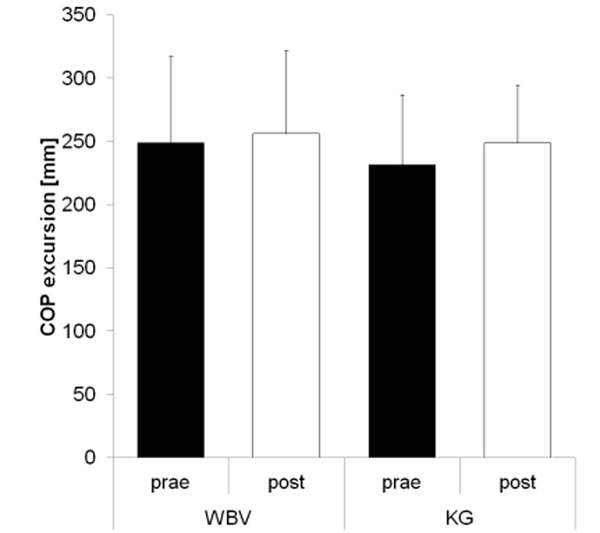
CoP excursions [mm]: right leg

No significant differences in vibration thresholds at all measured locations of both feet (fig. 1 shows data for the right Hallux) were found after WBV training for both groups. Similar results were evaluated for CoP excursions measured during standing with the right (fig. 2) and left legs.

## Conclusions

Whereas short term WBV training is shown to reduce plantar foot sensitivity but increase balance control [2], no effects of long term training could be seen. This may be due to adaptation effects to the linear, above-threshold stimulation characteristics of this kind of training. WBV seems not to be an adequate strategy to improve foot sensitivity or balance control of young healthy subjects.
